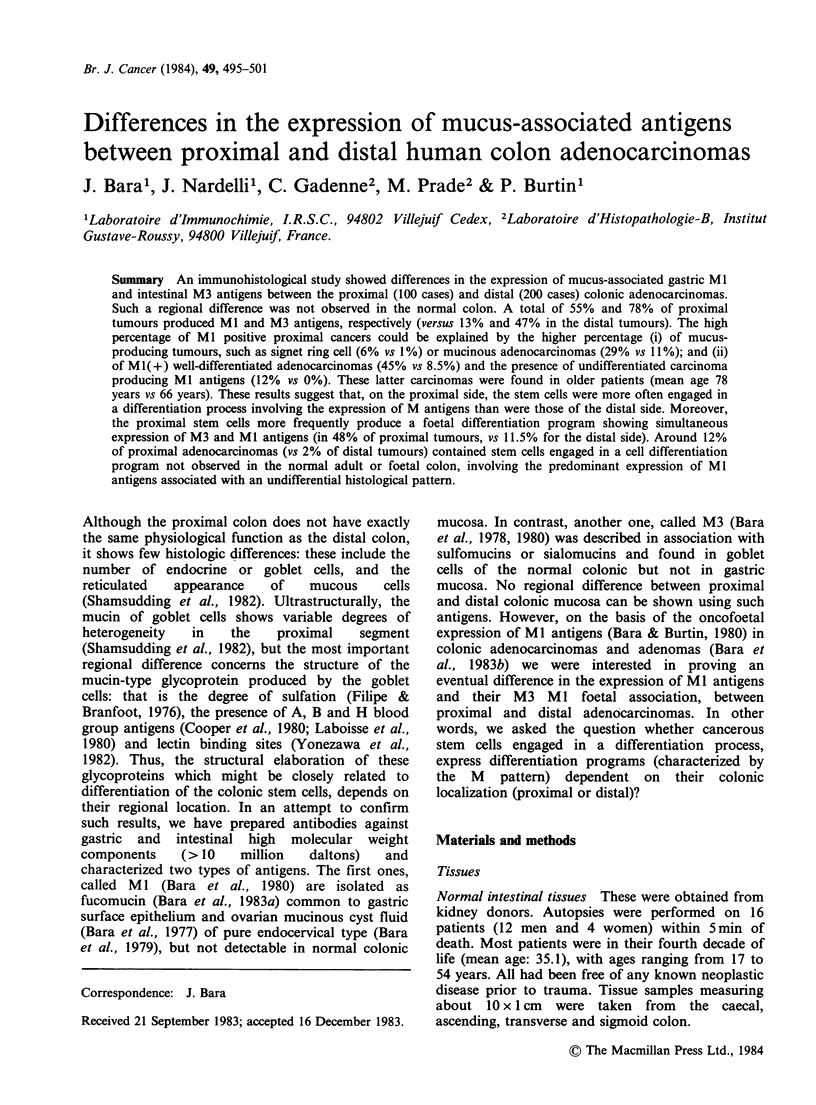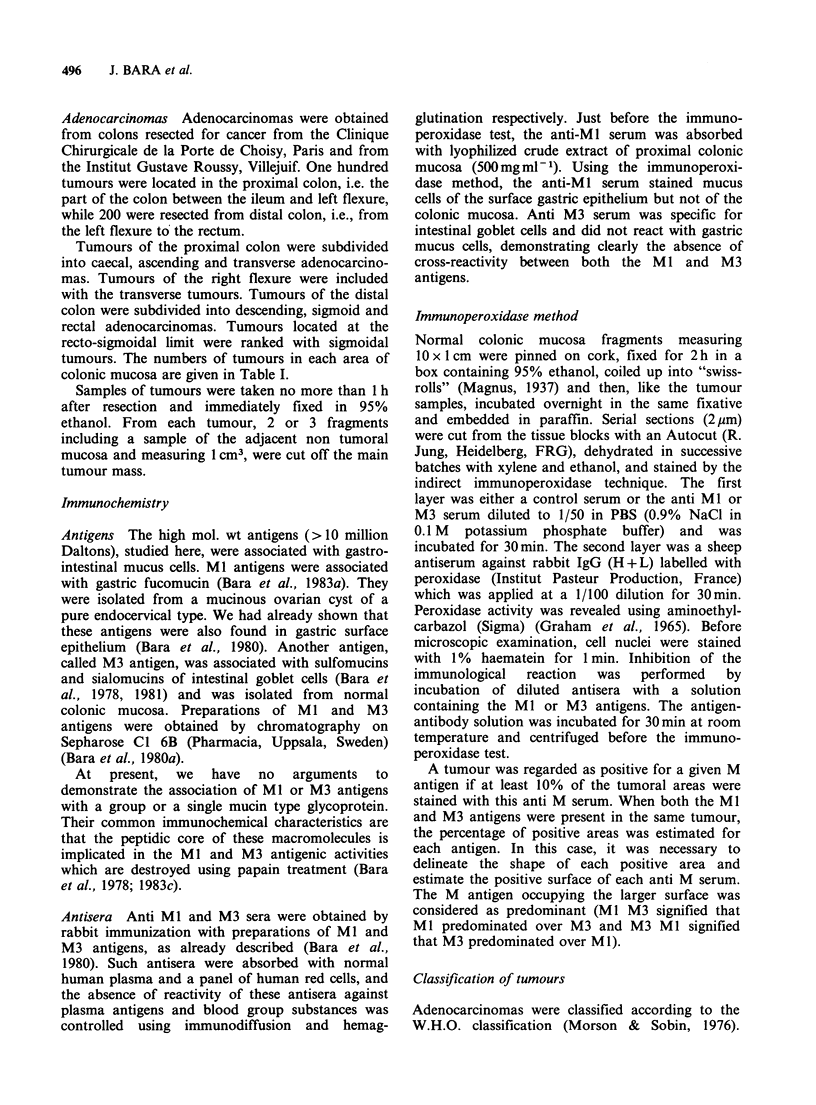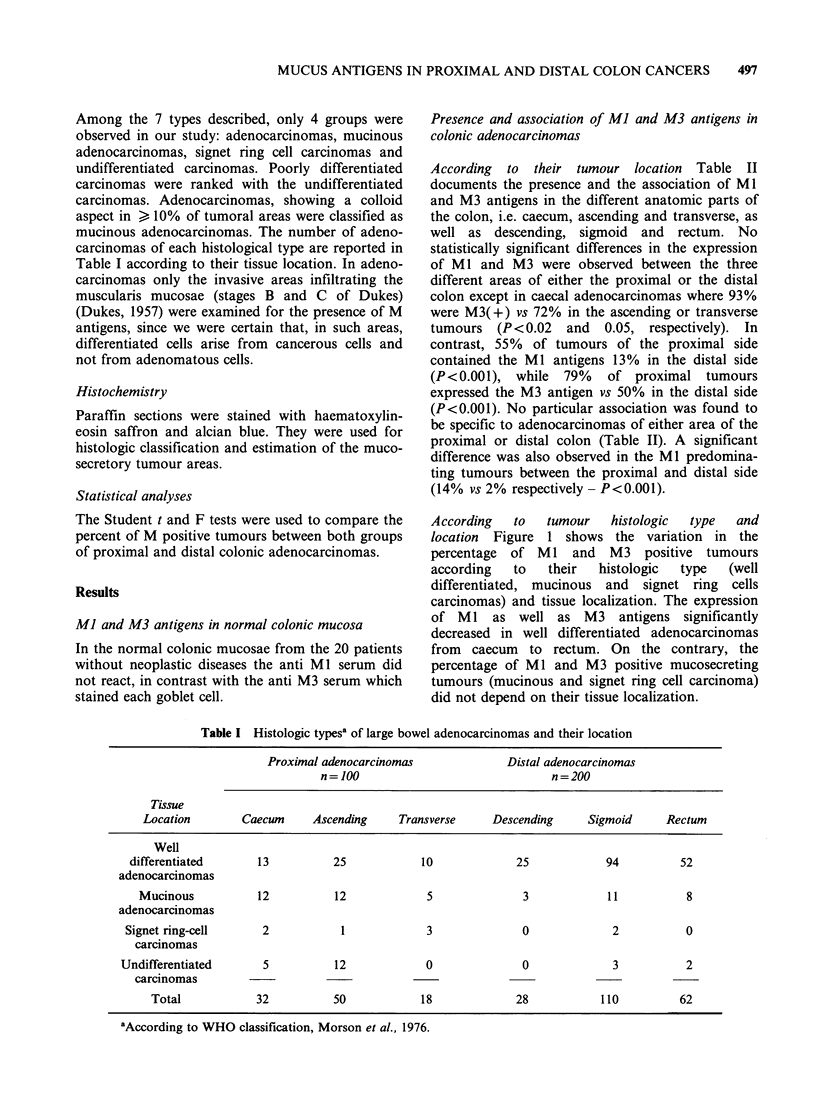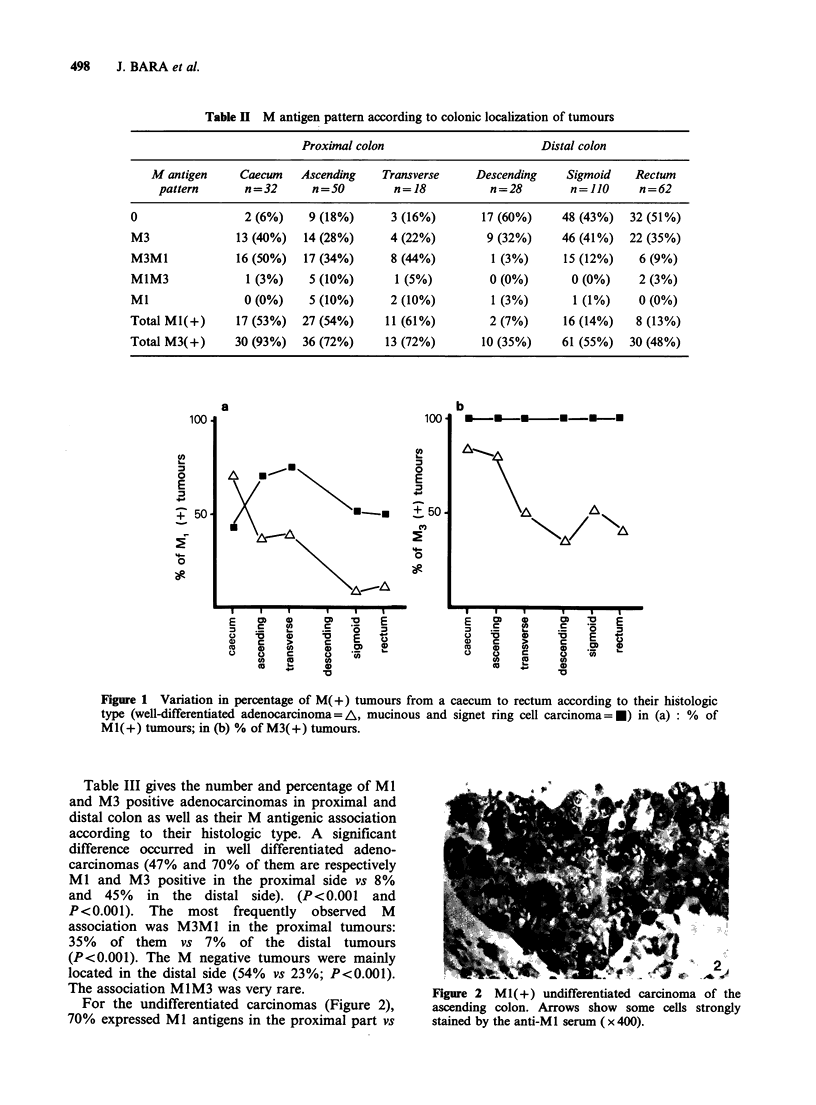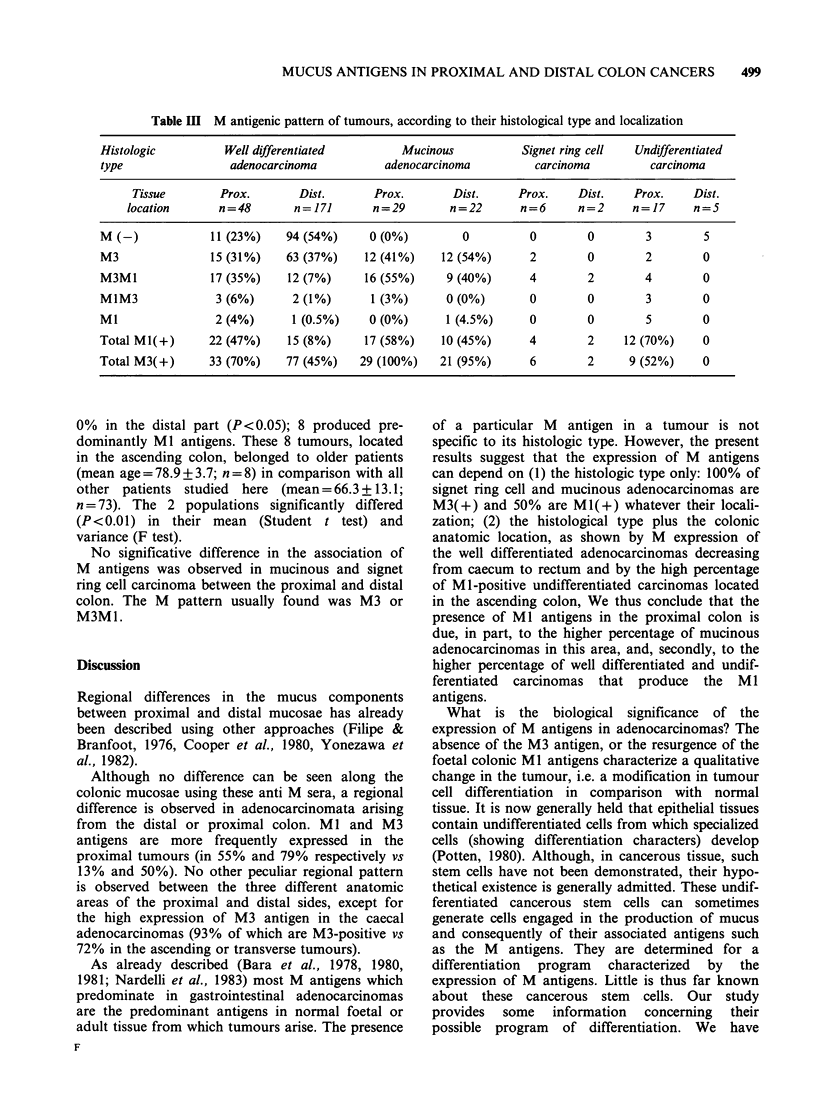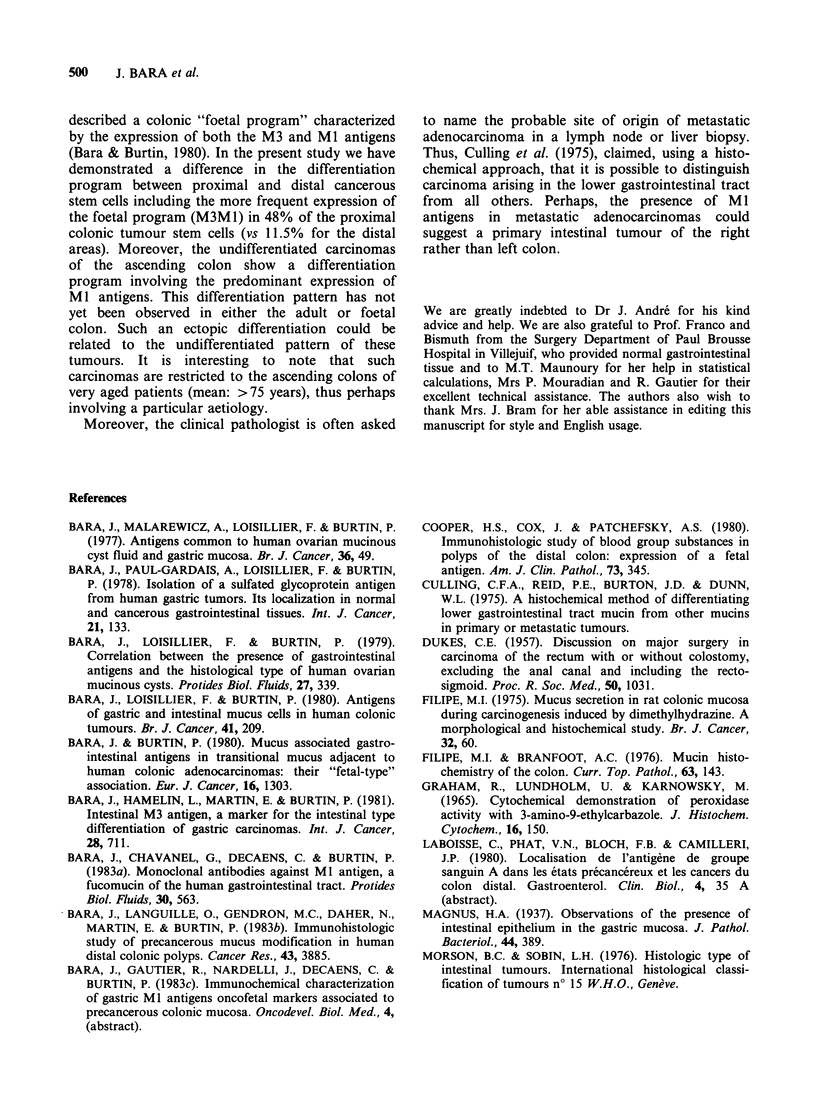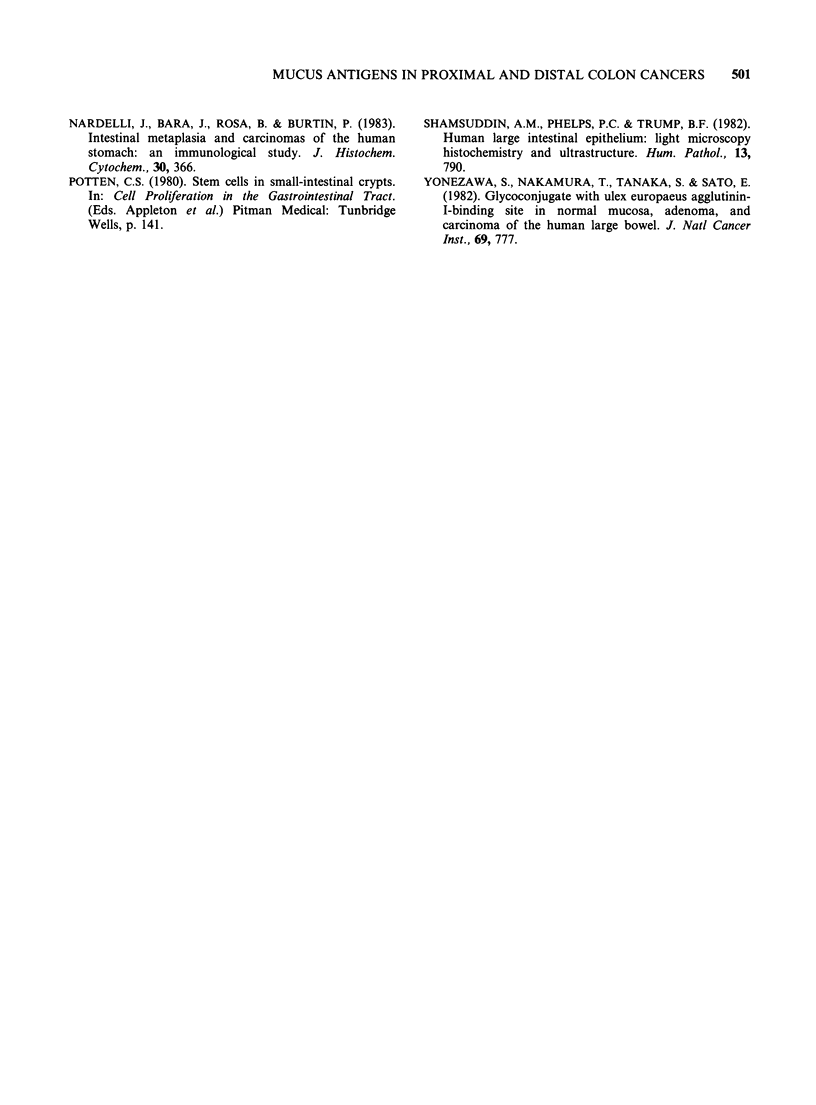# Differences in the expression of mucus-associated antigens between proximal and distal human colon adenocarcinomas.

**DOI:** 10.1038/bjc.1984.77

**Published:** 1984-04

**Authors:** J. Bara, J. Nardelli, C. Gadenne, M. Prade, P. Burtin

## Abstract

**Images:**


Br. J. Cancer (1984), **49**, 495–501

### Differences in the expression of mucus-associated antigens between proximal and distal human colon adenocarcinomas

J. Bara[1], J. Nardelli[1], C. Gadenne[2], M. Prade[2] & P. Burtin[1]

[1]*Laboratoire d'Immunochimie, I.R.S.C., 94802 Villejuif Cedex,* [2]*Laboratoire d'Histopathologie-B, Institut Gustave-Roussy, 94800 Villejuif, France.*

**Summary** An immunohistological study showed differences in the expression of mucus-associated gastric M1 and intestinal M3 antigens between the proximal (100 cases) and distal (200 cases) colonic adenocarcinomas. Such a regional difference was not observed in the normal colon. A total of 55% and 78% of proximal tumours produced M1 and M3 antigens, respectively (*versus* 13% and 47% in the distal tumours). The high percentage of M1 positive proximal cancers could be explained by the higher percentage (i) of mucus-producing tumours, such as signet ring cell (6% *vs* 1%) or mucinous adenocarcinomas (29% *vs* 11%); and (ii) of M1(+) well-differentiated adenocarcinomas (45% *vs* 8.5%) and the presence of undifferentiated carcinoma producing M1 antigens (12% *vs* 0%). These latter carcinomas were found in older patients (mean age 78 years *vs* 66 years). These results suggest that, on the proximal side, the stem cells were more often engaged in a differentiation process involving the expression of M antigens than were those of the distal side. Moreover, the proximal stem cells more frequently produce a foetal differentiation program showing simultaneous expression of M3 and M1 antigens (in 48% of proximal tumours, *vs* 11.5% for the distal side). Around 12% of proximal adenocarcinomas (*vs* 2% of distal tumours) contained stem cells engaged in a cell differentiation program not observed in the normal adult or foetal colon, involving the predominant expression of M1 antigens associated with an undifferential histological pattern.

Although the proximal colon does not have exactly the same physiological function as the distal colon, it shows few histologic differences: these include the number of endocrine or goblet cells, and the reticulated appearance of mucous cells (Shamsudding *et al.*, 1982). Ultrastructurally, the mucin of goblet cells shows variable degrees of heterogeneity in the proximal segment (Shamsudding *et al.*, 1982), but the most important regional difference concerns the structure of the mucin-type glycoprotein produced by the goblet cells: that is the degree of sulfation (Filipe & Branfoot, 1976), the presence of A, B and H blood group antigens (Cooper *et al.*, 1980; Laboisse *et al.*, 1980) and lectin binding sites (Yonezawa *et al.*, 1982). Thus, the structural elaboration of these glycoproteins which might be closely related to differentiation of the colonic stem cells, depends on their regional location. In an attempt to confirm such results, we have prepared antibodies against gastric and intestinal high molecular weight components (>10 million daltons) and characterized two types of antigens. The first ones, called M1 (Bara *et al.*, 1980) are isolated as fucomucin (Bara *et al.*, 1983*a*) common to gastric surface epithelium and ovarian mucinous cyst fluid (Bara *et al.*, 1977) of pure endocervical type (Bara *et al.*, 1979), but not detectable in normal colonic mucosa. In contrast, another one, called M3 (Bara *et al.*, 1978, 1980) was described in association with sulfomucins or sialomucins and found in goblet cells of the normal colonic but not in gastric mucosa. No regional difference between proximal and distal colonic mucosa can be shown using such antigens. However, on the basis of the oncofoetal expression of M1 antigens (Bara & Burtin, 1980) in colonic adenocarcinomas and adenomas (Bara *et al.*, 1983*b*) we were interested in proving an eventual difference in the expression of M1 antigens and their M3 M1 foetal association, between proximal and distal adenocarcinomas. In other words, we asked the question whether cancerous stem cells engaged in a differentiation process, express differentiation programs (characterized by the M pattern) dependent on their colonic localization (proximal or distal)?

Correspondence: J. Bara

Received 21 September 1983; accepted 16 December 1983.

### Materials and methods

### *Tissues*

*Normal intestinal tissues* These were obtained from kidney donors. Autopsies were performed on 16 patients (12 men and 4 women) within 5 min of death. Most patients were in their fourth decade of life (mean age: 35.1), with ages ranging from 17 to 54 years. All had been free of any known neoplastic disease prior to trauma. Tissue samples measuring about 10×1 cm were taken from the caecal, ascending, transverse and sigmoid colon.

© The Macmillan Press Ltd., 1984

*Adenocarcinomas* Adenocarcinomas were obtained from colons resected for cancer from the Clinique Chirurgicale de la Porte de Choisy, Paris and from the Institut Gustave Roussy, Villejuif. One hundred tumours were located in the proximal colon, i.e. the part of the colon between the ileum and left flexure, while 200 were resected from distal colon, i.e., from the left flexure to the rectum.

Tumours of the proximal colon were subdivided into caecal, ascending and transverse adenocarcinomas. Tumours of the right flexure were included with the transverse tumours. Tumours of the distal colon were subdivided into descending, sigmoid and rectal adenocarcinomas. Tumours located at the recto-sigmoidal limit were ranked with sigmoidal tumours. The numbers of tumours in each area of colonic mucosa are given in Table I.

Samples of tumours were taken no more than 1 h after resection and immediately fixed in 95% ethanol. From each tumour, 2 or 3 fragments including a sample of the adjacent non tumoral mucosa and measuring 1 cm$^3$, were cut off the main tumour mass.

*Immunochemistry*

*Antigens* The high mol. wt antigens (>10 million Daltons), studied here, were associated with gastro-intestinal mucus cells. M1 antigens were associated with gastric fucomucin (Bara *et al.*, 1983*a*). They were isolated from a mucinous ovarian cyst of a pure endocervical type. We had already shown that these antigens were also found in gastric surface epithelium (Bara *et al.*, 1980). Another antigen, called M3 antigen, was associated with sulfomucins and sialomucins of intestinal goblet cells (Bara *et al.*, 1978, 1981) and was isolated from normal colonic mucosa. Preparations of M1 and M3 antigens were obtained by chromatography on Sepharose Cl 6B (Pharmacia, Uppsala, Sweden) (Bara *et al.*, 1980*a*).

At present, we have no arguments to demonstrate the association of M1 or M3 antigens with a group or a single mucin type glycoprotein. Their common immunochemical characteristics are that the peptidic core of these macromolecules is implicated in the M1 and M3 antigenic activities which are destroyed using papain treatment (Bara *et al.*, 1978; 1983*c*).

*Antisera* Anti M1 and M3 sera were obtained by rabbit immunization with preparations of M1 and M3 antigens, as already described (Bara *et al.*, 1980). Such antisera were absorbed with normal human plasma and a panel of human red cells, and the absence of reactivity of these antisera against plasma antigens and blood group substances was controlled using immunodiffusion and hemagglutination respectively. Just before the immunoperoxidase test, the anti-M1 serum was absorbed with lyophilized crude extract of proximal colonic mucosa (500 mg ml$^{-1}$). Using the immunoperoxidase method, the anti-M1 serum stained mucus cells of the surface gastric epithelium but not of the colonic mucosa. Anti M3 serum was specific for intestinal goblet cells and did not react with gastric mucus cells, demonstrating clearly the absence of cross-reactivity between both the M1 and M3 antigens.

*Immunoperoxidase method*

Normal colonic mucosa fragments measuring 10 × 1 cm were pinned on cork, fixed for 2 h in a box containing 95% ethanol, coiled up into "swiss-rolls" (Magnus, 1937) and then, like the tumour samples, incubated overnight in the same fixative and embedded in paraffin. Serial sections (2 $\mu$m) were cut from the tissue blocks with an Autocut (R. Jung, Heidelberg, FRG), dehydrated in successive batches with xylene and ethanol, and stained by the indirect immunoperoxidase technique. The first layer was either a control serum or the anti M1 or M3 serum diluted to 1/50 in PBS (0.9% NaCl in 0.1 M potassium phosphate buffer) and was incubated for 30 min. The second layer was a sheep antiserum against rabbit IgG (H+L) labelled with peroxidase (Institut Pasteur Production, France) which was applied at a 1/100 dilution for 30 min. Peroxidase activity was revealed using aminoethylcarbazol (Sigma) (Graham *et al.*, 1965). Before microscopic examination, cell nuclei were stained with 1% haematein for 1 min. Inhibition of the immunological reaction was performed by incubation of diluted antisera with a solution containing the M1 or M3 antigens. The antigen-antibody solution was incubated for 30 min at room temperature and centrifuged before the immunoperoxidase test.

A tumour was regarded as positive for a given M antigen if at least 10% of the tumoral areas were stained with this anti M serum. When both the M1 and M3 antigens were present in the same tumour, the percentage of positive areas was estimated for each antigen. In this case, it was necessary to delineate the shape of each positive area and estimate the positive surface of each anti M serum. The M antigen occupying the larger surface was considered as predominant (M1 M3 signified that M1 predominated over M3 and M3 M1 signified that M3 predominated over M1).

*Classification of tumours*

Adenocarcinomas were classified according to the W.H.O. classification (Morson & Sobin, 1976).

Among the 7 types described, only 4 groups were observed in our study: adenocarcinomas, mucinous adenocarcinomas, signet ring cell carcinomas and undifferentiated carcinomas. Poorly differentiated carcinomas were ranked with the undifferentiated carcinomas. Adenocarcinomas, showing a colloid aspect in $\geqslant 10\%$ of tumoral areas were classified as mucinous adenocarcinomas. The number of adenocarcinomas of each histological type are reported in Table I according to their tissue location. In adenocarcinomas only the invasive areas infiltrating the muscularis mucosae (stages B and C of Dukes) (Dukes, 1957) were examined for the presence of M antigens, since we were certain that, in such areas, differentiated cells arise from cancerous cells and not from adenomatous cells.

*Histochemistry*

Paraffin sections were stained with haematoxylin-eosin saffron and alcian blue. They were used for histologic classification and estimation of the mucosecretory tumour areas.

*Statistical analyses*

The Student *t* and F tests were used to compare the percent of M positive tumours between both groups of proximal and distal colonic adenocarcinomas.

### Results

*M1 and M3 antigens in normal colonic mucosa*

In the normal colonic mucosae from the 20 patients without neoplastic diseases the anti M1 serum did not react, in contrast with the anti M3 serum which stained each goblet cell.

*Presence and association of M1 and M3 antigens in colonic adenocarcinomas*

*According to their tumour location* Table II documents the presence and the association of M1 and M3 antigens in the different anatomic parts of the colon, i.e. caecum, ascending and transverse, as well as descending, sigmoid and rectum. No statistically significant differences in the expression of M1 and M3 were observed between the three different areas of either the proximal or the distal colon except in caecal adenocarcinomas where 93% were M3(+) *vs* 72% in the ascending or transverse tumours ($P<0.02$ and 0.05, respectively). In contrast, 55% of tumours of the proximal side contained the M1 antigens 13% in the distal side ($P<0.001$), while 79% of proximal tumours expressed the M3 antigen *vs* 50% in the distal side ($P<0.001$). No particular association was found to be specific to adenocarcinomas of either area of the proximal or distal colon (Table II). A significant difference was also observed in the M1 predominating tumours between the proximal and distal side (14% *vs* 2% respectively – $P<0.001$).

*According to tumour histologic type and location* Figure 1 shows the variation in the percentage of M1 and M3 positive tumours according to their histologic type (well differentiated, mucinous and signet ring cells carcinomas) and tissue localization. The expression of M1 as well as M3 antigens significantly decreased in well differentiated adenocarcinomas from caecum to rectum. On the contrary, the percentage of M1 and M3 positive mucosecreting tumours (mucinous and signet ring cell carcinoma) did not depend on their tissue localization.

**Table I** Histologic types[a] of large bowel adenocarcinomas and their location

| | *Proximal adenocarcinomas n=100* | | | *Distal adenocarcinomas n=200* | | |
|---|---|---|---|---|---|---|
| *Tissue Location* | *Caecum* | *Ascending* | *Transverse* | *Descending* | *Sigmoid* | *Rectum* |
| Well differentiated adenocarcinomas | 13 | 25 | 10 | 25 | 94 | 52 |
| Mucinous adenocarcinomas | 12 | 12 | 5 | 3 | 11 | 8 |
| Signet ring-cell carcinomas | 2 | 1 | 3 | 0 | 2 | 0 |
| Undifferentiated carcinomas | 5 | 12 | 0 | 0 | 3 | 2 |
| Total | 32 | 50 | 18 | 28 | 110 | 62 |

[a]According to WHO classification, Morson *et al.*, 1976.

**Table II** M antigen pattern according to colonic localization of tumours

| | *Proximal colon* | | | *Distal colon* | | |
|---|---|---|---|---|---|---|
| *M antigen pattern* | *Caecum n=32* | *Ascending n=50* | *Transverse n=18* | *Descending n=28* | *Sigmoid n=110* | *Rectum n=62* |
| 0 | 2 (6%) | 9 (18%) | 3 (16%) | 17 (60%) | 48 (43%) | 32 (51%) |
| M3 | 13 (40%) | 14 (28%) | 4 (22%) | 9 (32%) | 46 (41%) | 22 (35%) |
| M3M1 | 16 (50%) | 17 (34%) | 8 (44%) | 1 (3%) | 15 (12%) | 6 (9%) |
| M1M3 | 1 (3%) | 5 (10%) | 1 (5%) | 0 (0%) | 0 (0%) | 2 (3%) |
| M1 | 0 (0%) | 5 (10%) | 2 (10%) | 1 (3%) | 1 (1%) | 0 (0%) |
| Total M1(+) | 17 (53%) | 27 (54%) | 11 (61%) | 2 (7%) | 16 (14%) | 8 (13%) |
| Total M3(+) | 30 (93%) | 36 (72%) | 13 (72%) | 10 (35%) | 61 (55%) | 30 (48%) |

**Figure 1** Variation in percentage of M(+) tumours from a caecum to rectum according to their histologic type (well-differentiated adenocarcinoma = △, mucinous and signet ring cell carcinoma = ■) in (a) : % of M1(+) tumours; in (b) % of M3(+) tumours.

Table III gives the number and percentage of M1 and M3 positive adenocarcinomas in proximal and distal colon as well as their M antigenic association according to their histologic type. A significant difference occurred in well differentiated adenocarcinomas (47% and 70% of them are respectively M1 and M3 positive in the proximal side *vs* 8% and 45% in the distal side). ($P<0.001$ and $P<0.001$). The most frequently observed M association was M3M1 in the proximal tumours: 35% of them *vs* 7% of the distal tumours ($P<0.001$). The M negative tumours were mainly located in the distal side (54% *vs* 23%; $P<0.001$). The association M1M3 was very rare.

For the undifferentiated carcinomas (Figure 2), 70% expressed M1 antigens in the proximal part *vs*

**Figure 2** M1(+) undifferentiated carcinoma of the ascending colon. Arrows show some cells strongly stained by the anti-M1 serum (×400).

**Table III** M antigenic pattern of tumours, according to their histological type and localization

| *Histologic type* | *Well differentiated adenocarcinoma* | | *Mucinous adenocarcinoma* | | *Signet ring cell carcinoma* | | *Undifferentiated carcinoma* | |
|---|---|---|---|---|---|---|---|---|
| *Tissue location* | *Prox. n=48* | *Dist. n=171* | *Prox. n=29* | *Dist. n=22* | *Prox. n=6* | *Dist. n=2* | *Prox. n=17* | *Dist. n=5* |
| M (−) | 11 (23%) | 94 (54%) | 0 (0%) | 0 | 0 | 0 | 3 | 5 |
| M3 | 15 (31%) | 63 (37%) | 12 (41%) | 12 (54%) | 2 | 0 | 2 | 0 |
| M3M1 | 17 (35%) | 12 (7%) | 16 (55%) | 9 (40%) | 4 | 2 | 4 | 0 |
| M1M3 | 3 (6%) | 2 (1%) | 1 (3%) | 0 (0%) | 0 | 0 | 3 | 0 |
| M1 | 2 (4%) | 1 (0.5%) | 0 (0%) | 1 (4.5%) | 0 | 0 | 5 | 0 |
| Total M1(+) | 22 (47%) | 15 (8%) | 17 (58%) | 10 (45%) | 4 | 2 | 12 (70%) | 0 |
| Total M3(+) | 33 (70%) | 77 (45%) | 29 (100%) | 21 (95%) | 6 | 2 | 9 (52%) | 0 |

0% in the distal part ($P<0.05$); 8 produced predominantly M1 antigens. These 8 tumours, located in the ascending colon, belonged to older patients (mean age$=78.9\pm3.7$; $n=8$) in comparison with all other patients studied here (mean$=66.3\pm13.1$; $n=73$). The 2 populations significantly differed ($P<0.01$) in their mean (Student *t* test) and variance (F test).

No significative difference in the association of M antigens was observed in mucinous and signet ring cell carcinoma between the proximal and distal colon. The M pattern usually found was M3 or M3M1.

### Discussion

Regional differences in the mucus components between proximal and distal mucosae has already been described using other approaches (Filipe & Branfoot, 1976, Cooper *et al.*, 1980, Yonezawa *et al.*, 1982).

Although no difference can be seen along the colonic mucosae using these anti M sera, a regional difference is observed in adenocarcinomata arising from the distal or proximal colon. M1 and M3 antigens are more frequently expressed in the proximal tumours (in 55% and 79% respectively *vs* 13% and 50%). No other peculiar regional pattern is observed between the three different anatomic areas of the proximal and distal sides, except for the high expression of M3 antigen in the caecal adenocarcinomas (93% of which are M3-positive *vs* 72% in the ascending or transverse tumours).

As already described (Bara *et al.*, 1978, 1980, 1981; Nardelli *et al.*, 1983) most M antigens which predominate in gastrointestinal adenocarcinomas are the predominant antigens in normal foetal or adult tissue from which tumours arise. The presence of a particular M antigen in a tumour is not specific to its histologic type. However, the present results suggest that the expression of M antigens can depend on (1) the histologic type only: 100% of signet ring cell and mucinous adenocarcinomas are M3(+) and 50% are M1(+) whatever their localization; (2) the histological type plus the colonic anatomic location, as shown by M expression of the well differentiated adenocarcinomas decreasing from caecum to rectum and by the high percentage of M1-positive undifferentiated carcinomas located in the ascending colon, We thus conclude that the presence of M1 antigens in the proximal colon is due, in part, to the higher percentage of mucinous adenocarcinomas in this area, and, secondly, to the higher percentage of well differentiated and undifferentiated carcinomas that produce the M1 antigens.

What is the biological significance of the expression of M antigens in adenocarcinomas? The absence of the M3 antigen, or the resurgence of the foetal colonic M1 antigens characterize a qualitative change in the tumour, i.e. a modification in tumour cell differentiation in comparison with normal tissue. It is now generally held that epithelial tissues contain undifferentiated cells from which specialized cells (showing differentiation characters) develop (Potten, 1980). Although, in cancerous tissue, such stem cells have not been demonstrated, their hypothetical existence is generally admitted. These undifferentiated cancerous stem cells can sometimes generate cells engaged in the production of mucus and consequently of their associated antigens such as the M antigens. They are determined for a differentiation program characterized by the expression of M antigens. Little is thus far known about these cancerous stem cells. Our study provides some information concerning their possible program of differentiation. We have described a colonic "foetal program" characterized by the expression of both the M3 and M1 antigens (Bara & Burtin, 1980). In the present study we have demonstrated a difference in the differentiation program between proximal and distal cancerous stem cells including the more frequent expression of the foetal program (M3M1) in 48% of the proximal colonic tumour stem cells (*vs* 11.5% for the distal areas). Moreover, the undifferentiated carcinomas of the ascending colon show a differentiation program involving the predominant expression of M1 antigens. This differentiation pattern has not yet been observed in either the adult or foetal colon. Such an ectopic differentiation could be related to the undifferentiated pattern of these tumours. It is interesting to note that such carcinomas are restricted to the ascending colons of very aged patients (mean: >75 years), thus perhaps involving a particular aetiology.

Moreover, the clinical pathologist is often asked to name the probable site of origin of metastatic adenocarcinoma in a lymph node or liver biopsy. Thus, Culling *et al.* (1975), claimed, using a histochemical approach, that it is possible to distinguish carcinoma arising in the lower gastrointestinal tract from all others. Perhaps, the presence of M1 antigens in metastatic adenocarcinomas could suggest a primary intestinal tumour of the right rather than left colon.

We are greatly indebted to Dr J. André for his kind advice and help. We are also grateful to Prof. Franco and Bismuth from the Surgery Department of Paul Brousse Hospital in Villejuif, who provided normal gastrointestinal tissue and to M.T. Maunoury for her help in statistical calculations, Mrs P. Mouradian and R. Gautier for their excellent technical assistance. The authors also wish to thank Mrs. J. Bram for her able assistance in editing this manuscript for style and English usage.

### References

BARA, J., MALAREWICZ, A., LOISILLIER, F. & BURTIN, P. (1977). Antigens common to human ovarian mucinous cyst fluid and gastric mucosa. *Br. J. Cancer*, **36**, 49.

BARA, J., PAUL-GARDAIS, A., LOISILLIER, F. & BURTIN, P. (1978). Isolation of a sulfated glycoprotein antigen from human gastric tumors. Its localization in normal and cancerous gastrointestinal tissues. *Int. J. Cancer*, **21**, 133.

BARA, J., LOISILLIER, F. & BURTIN, P. (1979). Correlation between the presence of gastrointestinal antigens and the histological type of human ovarian mucinous cysts. *Protides Biol. Fluids*, **27**, 339.

BARA, J., LOISILLIER, F. & BURTIN, P. (1980). Antigens of gastric and intestinal mucus cells in human colonic tumours. *Br. J. Cancer*, **41**, 209.

BARA, J. & BURTIN, P. (1980). Mucus associated gastrointestinal antigens in transitional mucus adjacent to human colonic adenocarcinomas: their "fetal-type" association. *Eur. J. Cancer*, **16**, 1303.

BARA, J., HAMELIN, L., MARTIN, E. & BURTIN, P. (1981). Intestinal M3 antigen, a marker for the intestinal type differentiation of gastric carcinomas. *Int. J. Cancer*, **28**, 711.

BARA, J., CHAVANEL, G., DECAENS, C. & BURTIN, P. (1983*a*). Monoclonal antibodies against M1 antigen, a fucomucin of the human gastrointestinal tract. *Protides Biol. Fluids*, **30**, 563.

BARA, J., LANGUILLE, O., GENDRON, M.C., DAHER, N., MARTIN, E. & BURTIN, P. (1983*b*). Immunohistologic study of precancerous mucus modification in human distal colonic polyps. *Cancer Res.*, **43**, 3885.

BARA, J., GAUTIER, R., NARDELLI, J., DECAENS, C. & BURTIN, P. (1983*c*). Immunochemical characterization of gastric M1 antigens oncofetal markers associated to precancerous colonic mucosa. *Oncodevel. Biol. Med.*, **4**, (abstract).

COOPER, H.S., COX, J. & PATCHEFSKY, A.S. (1980). Immunohistologic study of blood group substances in polyps of the distal colon: expression of a fetal antigen. *Am. J. Clin. Pathol.*, **73**, 345.

CULLING, C.F.A., REID, P.E., BURTON, J.D. & DUNN, W.L. (1975). A histochemical method of differentiating lower gastrointestinal tract mucin from other mucins in primary or metastatic tumours.

DUKES, C.E. (1957). Discussion on major surgery in carcinoma of the rectum with or without colostomy, excluding the anal canal and including the recto-sigmoid. *Proc. R. Soc. Med.*, **50**, 1031.

FILIPE, M.I. (1975). Mucus secretion in rat colonic mucosa during carcinogenesis induced by dimethylhydrazine. A morphological and histochemical study. *Br. J. Cancer*, **32**, 60.

FILIPE, M.I. & BRANFOOT, A.C. (1976). Mucin histochemistry of the colon. *Curr. Top. Pathol.*, **63**, 143.

GRAHAM, R., LUNDHOLM, U. & KARNOWSKY, M. (1965). Cytochemical demonstration of peroxidase activity with 3-amino-9-ethylcarbazole. *J. Histochem. Cytochem.*, **16**, 150.

LABOISSE, C., PHAT, V.N., BLOCH, F.B. & CAMILLERI, J.P. (1980). Localisation de l'antigène de groupe sanguin A dans les états précancéreux et les cancers du colon distal. Gastroenterol. *Clin. Biol.*, **4**, 35 A (abstract).

MAGNUS, H.A. (1937). Observations of the presence of intestinal epithelium in the gastric mucosa. *J. Pathol. Bacteriol.*, **44**, 389.

MORSON, B.C. & SOBIN, L.H. (1976). Histologic type of intestinal tumours. International histological classification of tumours n° 15 *W.H.O., Genève*.

NARDELLI, J., BARA, J., ROSA, B. & BURTIN, P. (1983). Intestinal metaplasia and carcinomas of the human stomach: an immunological study. *J. Histochem. Cytochem.*, **30**, 366.

POTTEN, C.S. (1980). Stem cells in small-intestinal crypts. In: *Cell Proliferation in the Gastrointestinal Tract*. (Eds. Appleton *et al.*) Pitman Medical: Tunbridge Wells, p. 141.

SHAMSUDDIN, A.M., PHELPS, P.C. & TRUMP, B.F. (1982). Human large intestinal epithelium: light microscopy histochemistry and ultrastructure. *Hum. Pathol.*, **13**, 790.

YONEZAWA, S., NAKAMURA, T., TANAKA, S. & SATO, E. (1982). Glycoconjugate with ulex europaeus agglutinin-I-binding site in normal mucosa, adenoma, and carcinoma of the human large bowel. *J. Natl Cancer Inst.*, **69**, 777.